# Efficacy of endovascular treatment for completely occlusive acute–subacute portal and mesenteric vein thrombosis with severe complications in patients without cirrhosis

**DOI:** 10.1007/s11604-022-01377-9

**Published:** 2023-01-21

**Authors:** Hidemasa Saito, Fumie Sugihara, Tatsuo Ueda, Hiromitsu Hayashi, Sayaka Shirai, Taiga Matsumoto, Ryutaro Fujitsuna, Shin-ichiro Kumita

**Affiliations:** Department of Radiology, Nippon Medical School, 1-1-5, Sendagi, Bunkyo-ku, Tokyo, 113-8603 Japan

**Keywords:** Portal vein, Venous thrombosis, Angioplasty, Thrombectomy

## Abstract

**Purpose:**

Completely occlusive acute–subacute portal and mesenteric vein thrombosis (PVMVT) with severe complications is fatal. Endovascular treatments (EVTs) of acute–subacute PVMVT are not standardized. Thrombectomy combined with continuous catheter-directed thrombolysis is considered an effective treatment. Here, we aimed to evaluate the outcome of EVTs of completely occlusive acute–subacute PVMVT with severe complications in patients without cirrhosis.

**Materials and methods:**

Nineteen patients (nine men and 10 women; age, 60.1 ± 16.8 years) with completely occlusive acute–subacute PVMVT were retrospectively assessed. Acute–subacute PVMVT was defined as symptom onset within 40 days, with no cavernous transformation observed on contrast-enhanced computed tomography. The patients were treated with EVTs, a combination of thrombectomy (including aspiration thrombectomy, plain old balloon angioplasty, single injection of thrombolytic agents, and stent placement) and continuous catheter-directed thrombolysis. Kaplan–Meier analyses were performed to assess all-cause mortality, acute–subacute PVMVT-related mortality, and portal vein (PV) patency. The degree of recanalization and patency of PV, complications, factors related to acute–subacute PVMVT-related mortality, and factors related to patency of PV were also evaluated.

**Results:**

The all-cause and acute–subacute PVMVT-related mortality rates were 36.8% (7/19) and 31.6% (6/19), respectively. Seven (36.8%) and 11 (57.9%) patients achieved complete and partial recanalization, respectively. Among the 18 patients who achieved recanalization, follow-up images after 608.7 ± 889.5 days confirmed recanalization in 83.3% (15/18) patients, and 53.3% (8/15) of these patients achieved patency of PV. Seven patients (36.8%) developed complications, and two (10.5%) required interventional treatment for complications. Deterioration of liver function significantly worsened the prognosis (*P* = 0.046), while anticoagulation therapy significantly maintained portal patency (*P* = 0.03).

**Conclusion:**

This endovascular method for acute–subacute PVMVT, which combines thrombectomy and continuous catheter-directed thrombolysis EVT approach was effective for thrombus resolution. However, further studies must define conditions that improve patient prognosis.

## Introduction

Portal vein (PV) and mesenteric vein (MV) thrombosis (PVMVT) is rare, with an incidence of approximately 0.7–2.7 per 100 000 individuals per year [1, 2]. The acute and subacute onset of PVMVT is even rarer; however, the specific incidence rates are unknown. Many cases of acute–subacute PVMVT remain asymptomatic, but serious, painful clinical courses, such as bowel necrosis, varicose vein bleeding associated with portal hypertension, and liver failure, with mortality rates of approximately 50–75%, have been reported [3–8].

The treatment of choice for PVMVT depends on the presenting symptoms and is thus difficult to determine, because some patients are asymptomatic, whereas others develop life-threatening complications. Systemic anticoagulation is the first choice of treatment for acute–subacute PVMVT and can reduce PVMVT to some extent; however, it is sometimes inefficient in severe acute–subacute PVMVT, especially in juvenile cases [5, 9–11]. Significant portal hypertension develops if PVMVT remains undissolved with systemic anticoagulation therapy [12]. Current management of acute–subacute PVMVT relies on early diagnosis, adequate resuscitation, and prompt initiation of systemic anticoagulation as per the 2009 American Association for the Study of Liver Diseases practice guidelines [13, 14]. However, these guidelines provide no recommendations for thrombolysis and utilize limited evidence from two retrospective surveys prior to 2000 and only one study (*n* = 10) for anticoagulation in non-cirrhotic PVMVT [13].

Recently, in addition to systemic anticoagulation, endovascular treatments (EVTs) including aspiration thrombectomy (AT), catheter-directed thrombolysis (CDT), plain old balloon angioplasty (POBA), and stent placement have been reported to be useful for PVMVT management [13, 15–22]. Immediate revascularization of EVTs to normalize the portal pressure has been proposed [23, 24]. However, they are yet to be established as recommended treatment strategies for this condition, and institutions utilize unique regimens that differ in indication, route of delivery, and technique.

A novel endovascular method for acute–subacute PVMVT is a combination of thrombectomy (including AT, POBA, a single-shot injection of thrombolytic agents, and stent placement) and continuous CDT. Studies and case reports on the efficacy of this method are limited [17, 25–28]. There are even fewer reports on completely occlusive acute–subacute PVMVT [25, 26, 28, 29]. Therefore, this study aimed to evaluate the efficacy and factors related to the outcome of EVTs for completely occlusive acute–subacute PVMVT (symptom onset < 40 days) with severe complications.

## Materials and methods

The protocol for this retrospective study was approved by the institutional review board of our institution (B-2021–493) and complies with the Health Insurance Portability and Accountability Act.

### Patients

**Fig. 1:**
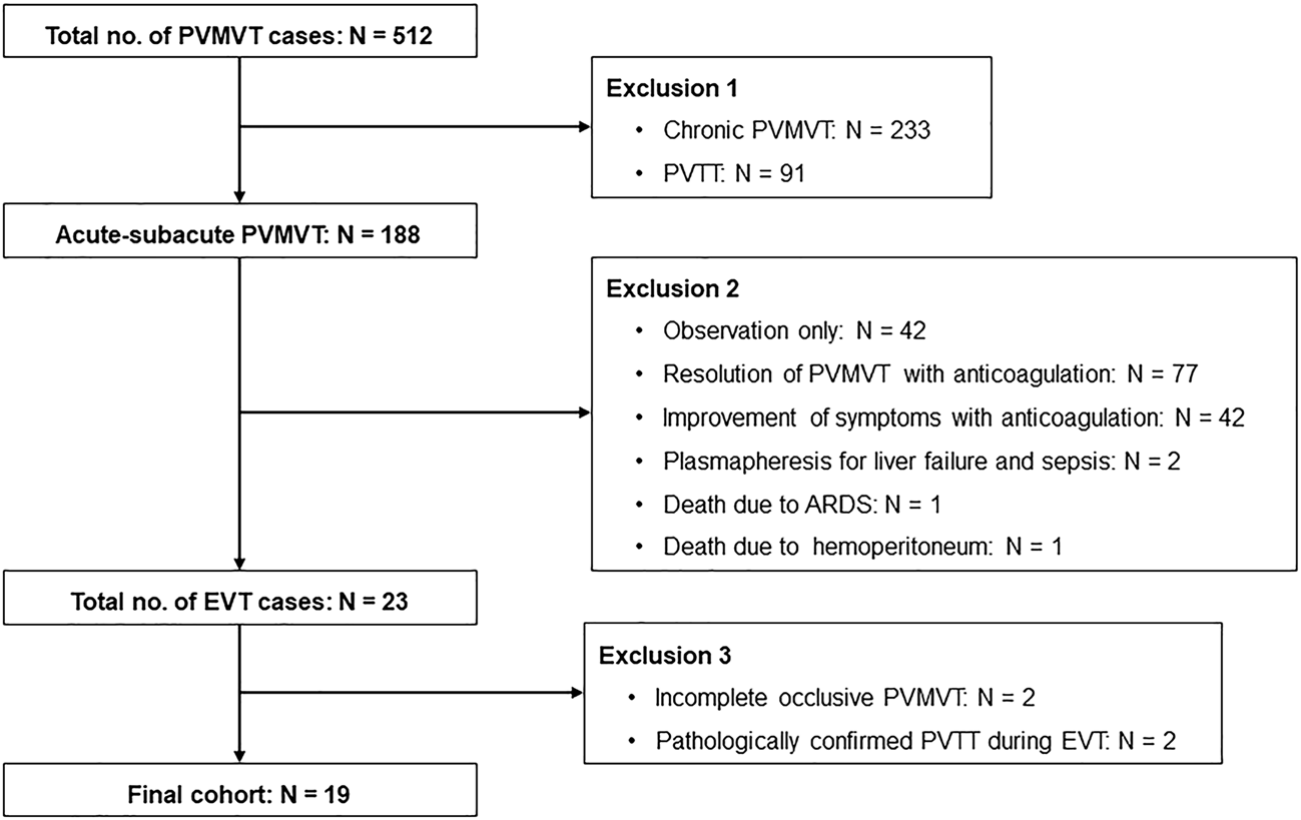
Flowchart of cohort construction. *ARDS* acute respiratory distress syndrome, *EVT* endovascular treatment, *PVMVT* portal and mesenteric vein thrombosis, *PVTT* portal venous tumor thrombosis

Figure 1 shows the flowchart of cohort construction for statistical analyses. Overall, 512 patients were diagnosed with PVMVT or portal venous tumor thrombosis (PVTT) at our institution using contrast-enhanced computed tomography (CECT) between April 2007 and January 2022. Acute–subacute PVMVT was diagnosed according to symptoms, and CECT was performed to confirm occlusive PVMVT before EVTs in all patients. Acute–subacute PVMVT was defined as the onset of symptoms, such as abdominal pain and fever, within 40 days, with no cavernous transformation observed on CECT. Completely occlusive PVMVT was defined as complete occlusion of either PV or MV.

All patients with symptomatic PVMVT were treated with systemic anticoagulation therapy initially. EVT indications for acute–subacute PVMVT were: (i) symptoms of bowel ischemia, (ii) worsening of liver function despite systemic anticoagulation, and (iii) expansion of PVMVT despite systemic anticoagulation. The exclusion criteria were: (i) incomplete occlusive PVMVT, (ii) pathologically confirmed PVTT during EVT, (iii) disseminated intravascular coagulation, and (iv) massive ascites.

A total of 188 patients were diagnosed with acute–subacute PVMVT, 91 were diagnosed with PVTT, and 233 were diagnosed with chronic PVMVT. Among patients with acute–subacute PVMVT, 42 asymptomatic patients were observed without systemic anticoagulation therapy, 1 died of acute respiratory distress syndrome after bowel resection, and 2 underwent plasmapheresis due to liver failure and sepsis. The remaining 120 patients underwent systemic anticoagulation therapy. Among patients who underwent systemic anticoagulation therapy, PVMVT resolved in 77, and PVMVT persisted in 42, but the symptoms improved. One patient who underwent systemic anticoagulation therapy died of hemoperitoneum. Twenty-three patients underwent EVTs during the study period. Two patients were excluded due to incomplete occlusive PVMVT, and two were excluded due to pathologically confirmed PVTT during EVT. The remaining 19 patients (nine men and 10 women; age range, 20–80 years; mean age, 60.1 ± 16.8 years) with completely occlusive acute–subacute PVMVT, who underwent EVTs, were enrolled in this study. The demographic data of the study population, associated etiologies, and EVT indications are presented in Table 1.

**Table 1:** Patient demographics

Age (years, mean ± SD; range)	60.1 ± 16.8; 20–80
Sex (male/female)	9/10
Indication*	
Bowel ischemia	8 (42.1%)
Liver dysfunction	7 (36.8%)
Expansion of PVMVT	9 (47.4%)
Etiology	
Biliary fistula	3 (15.8%)
Pancreatic fistula	2 (10.5%)
SMV stenosis due to resection of colon cancer	2 (10.5%)
PV stenosis due to liver transplantation	1 (5.3%)
Perforation of rectum and peritonitis due to polypectomy	1 (5.3%)
Protein C deficiency	3 (15.8%)
Antithrombin III deficiency	1 (5.3%)
Antiphospholipid syndrome	1 (5.3%)
Peritonitis due to colonic diverticulitis	2 (10.5%)
Amebic liver abscess	1 (5.3%)
Cholecystitis	1 (5.3%)

*PV* portal vein, *SD* standard deviation, *SMV* superior mesenteric vein thrombosis

*It contains duplicates

### Endovascular therapy

EVTs were performed via one of the following two access routes: the percutaneous transhepatic approach or transileocolic approach. In the percutaneous transhepatic approach, following percutaneous puncture of the intrahepatic portal branch under sonographic guidance, a catheter was advanced into the portal venous system. In the transileocolic approach, following the exposure of the distal ileum through a small abdominal incision, a catheter was advanced into the portal venous system via the ileocolic vein.

The percutaneous transhepatic approach was the first choice; however, the transileocolic approach was selected when bowel resection was necessary, and it was difficult to puncture the intrahepatic PV due to complete obstruction of both right and left hepatic PV. Bowel resection was performed before EVTs for patients with symptoms of bowel necrosis.

The endovascular method used in this study comprised a combination of the temporary EVT session and the continuous CDT. The endpoint of the combination therapy was recanalization of the intrahepatic PV of at least one lobe of the liver, main tract of the PV, and main tract of the superior mesenteric vein (SMV).

#### Temporary EVT session

An 8-Fr sheath (Supersheath; Medikit, Tokyo, Japan) was inserted into the portal venous system via the transhepatic or transileocolic route, and heparin was administered to maintain the activated clotting time within the range of 200–300 s during temporary EVT sessions. A 6-Fr catheter (Aspiraircass; Medikit) and an 8-Fr guiding catheter (Launcher; Medtronic, Dublin, Ireland) were used for manual AT. A one-shot injection of the thrombolytic agent was administered using urokinase (Mochida, Tokyo, Japan) via a 5-Fr multiple-side-hole infusion catheter (Fountain Infusion System; Merit Medical, South Jordan, UT, USA). POBA was performed for fragmentation of the thrombus and crimping the thrombus to the vessel wall. Bare metal stents (WALLSTENT; Boston Scientific, Marlborough, MA, US. E-LUMINEXX; BD, Tokyo, Japan) were used in cases with mechanical stenosis or thrombi refractory to AT, POBA, and CDT. Most cases were initiated with AT to remove the thrombus as much as possible. CDT and POBA were performed for residual thrombi refractory to AT. Primary stenting was performed in cases of mechanical stenosis and thrombus confined to the main trunk of the PV and SMV. The continuous CDT session was initialized when recanalization of PV and SMV was not achieved.

#### Continuous CDT

During the interval between each temporary EVT session, an 8-Fr sheath (Medikit) and 5-Fr heparin-coated catheter (Anthron; Toray Medical, Tokyo, Japan) remained in place within the SMV and PV. Continuous CDT using urokinase was performed via the sheath (120,000 U/day) and catheter (240,000 U/day) for 24–48 h until the next temporary EVT session. Continuous CDT via the ileocolic vein was managed using an open abdomen approach with vacuum-assisted wound closure (VAC) in the intensive care unit. This open abdomen approach was limited to a maximum of 7 days.

The procedural details are summarized in Table 2. The average number of temporary EVT sessions was 2.8 ± 3.1 (range, 1–15), and the average duration of continuous CDT was 4.8 ± 5.4 (range, 0–24) days. Eight patients did not undergo continuous CDT, as recanalization was achieved with one temporary EVT session. Five of the eight patients who presented with bowel ischemia symptoms required bowel resection due to bowel necrosis prior to EVTs. None of the patients received transplants after EVTs.

**Table 2:** Procedural details

Approach route	
Transhepatic	16 (84.2%)
Transileocolic	3 (15.8%)
No. temporary EVT sessions (mean ± SD; range)	2.8 ± 3.1; 1–15
Duration of continuous CDT (days, mean ± SD; range)	4.8 ± 5.4; 0–24
Dosage of urokinase (× 10000U, mean ± SD; range)	111.8 ± 128.3; 0–546
Temporary EVT procedure	
AT + CDT + POBA	6 (31.6%)
AT + CDT	4 (21.1%)
BMS + AT + CDT + POBA	3 (15.8%)
BMS + CDT	3 (15.8%)
BMS + POBA	2 (10.5%)
BMS + AT + CDT	1 (5.3%)
Bowel resection	
Bowel resection before the EVTs	5 (26.3%)
Bowel resection after the EVTs	2 (10.5%)

*AT* aspiration thrombectomy *BMS* bare metal stents, *CDT* catheter-directed thrombolysis, *EVT* endovascular treatment, *POBA* plain old balloon angioplasty, *SD* standard deviation

**Fig. 2:**
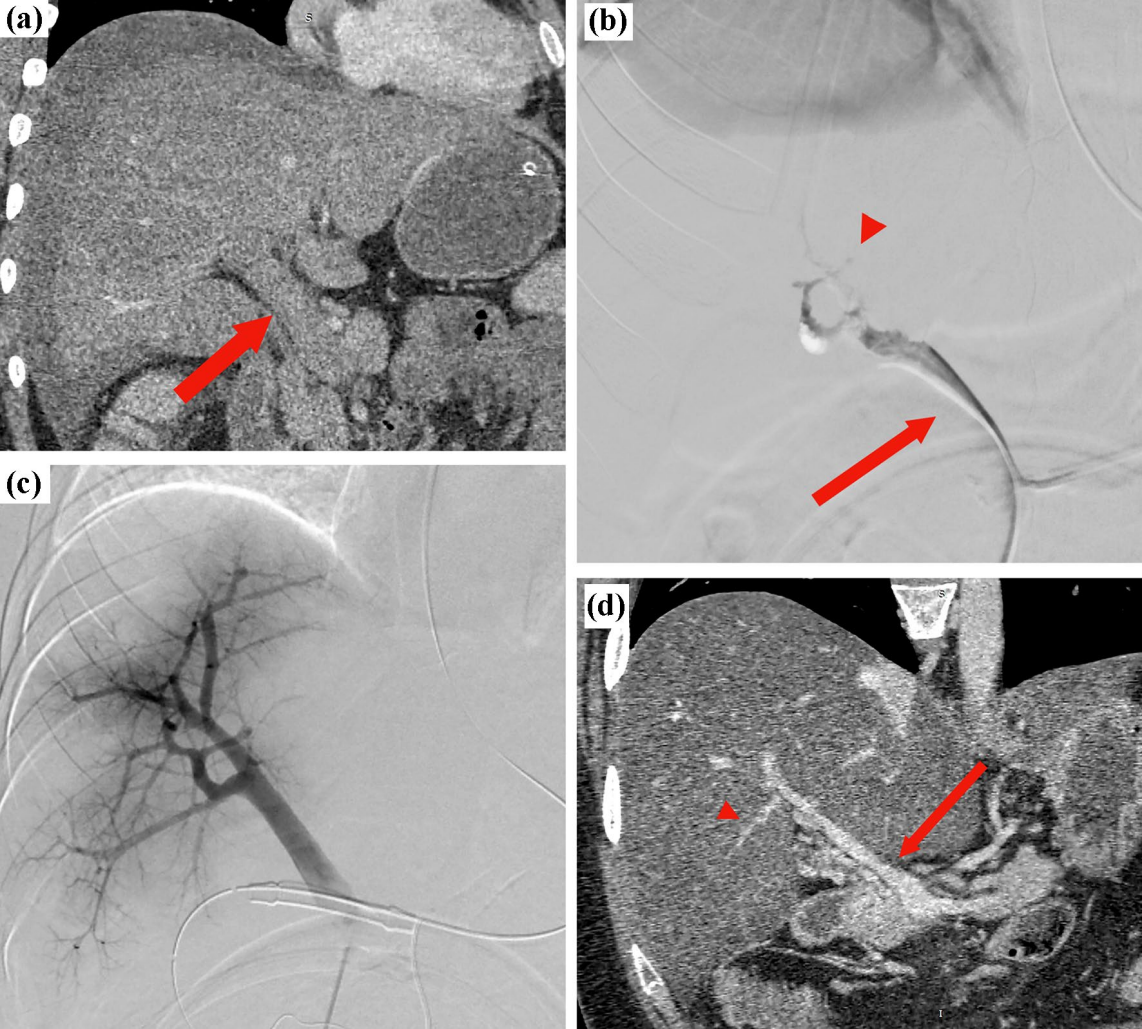
A representative case of the transileocolic approach. A male patient in his early twenties presented with completely occlusive acute portal and mesenteric vein thrombosis with bowel necrosis due to anti-phospholipid antibody syndrome. Transileocolic angioplasty was performed after necrotic bowel resection. **a** shows completely occluded main trunks of the portal (arrow). **b** shows the portography via the ileocolic vein. Portography showed completely occluded main trunks of the portal (arrow) and intrahepatic portal veins (arrowhead). **c** shows the portography after three sessions of temporary endovascular treatments (EVTs) and continuous catheter-directed thrombolysis via the ileocolic vein with open abdomen with vacuum-assisted wound closure in intensive care units. A partial recanalization was performed. **d** shows the contrast-enhanced computed tomography image obtained 2 years after EVTs. Patency of the main trunk of the portal vein (arrow) and intrahepatic portal veins (arrowhead) was achieved. *CDT* catheter-directed thrombolysis, *CECT* contrast-enhanced computed tomography, *EVT* endovascular treatment

Figure 2 shows a representative case of the transileocolic approach with bowel ischemia.

### Evaluation

The primary outcomes were all-cause mortality and acute–subacute PVMVT-related mortality throughout follow-up. The secondary outcomes were the degree of recanalization immediately after EVTs, patency of PV on follow-up CECT or sonography, complications, factors related to acute–subacute PVMVT-related mortality, and factors related to patency of PV.

The degree of recanalization was evaluated using venography after EVTs and was considered (i) complete if no intravascular material remained, and blood flow was restored, (ii) partial if the thrombosis was less extensive after intervention than before, or (iii) unchanged.

We evaluated complications using the Clavien–Dindo classification [30].

Factors related to acute–subacute PVMVT-related mortality were evaluated by EVT indication, etiology, EVT procedure, degree of recanalization, and patency of PV on follow-up CECT. Factors related to patency of PV were evaluated by EVT indication, etiology, EVT procedure, degree of recanalization, and oral anticoagulation after EVTs.

### Statistical analysis

All statistical analyses were performed using EZR (Saitama Medical Center, Saitama, Japan, version 1.54), a graphical user interface for R (The R Foundation for Statistical Computing, Vienna, Austria, version 4.0.3). It is a modified version of the R commander (version 2.7–1) designed to add frequently used statistical functions in biostatistics [31]. Continuous variables are presented as the mean ± standard deviation, whereas categorical data are presented as percentages. Kaplan–Meier analyses were performed to assess all-cause mortality, acute–subacute PVMVT-related mortality, and patency of PV on follow-up CECT or sonography. Categorical variables were compared using Fisher’s exact test. A P value < 0.05 was considered statistically significant.

## Results

### Overall survival

**Fig. 3:**
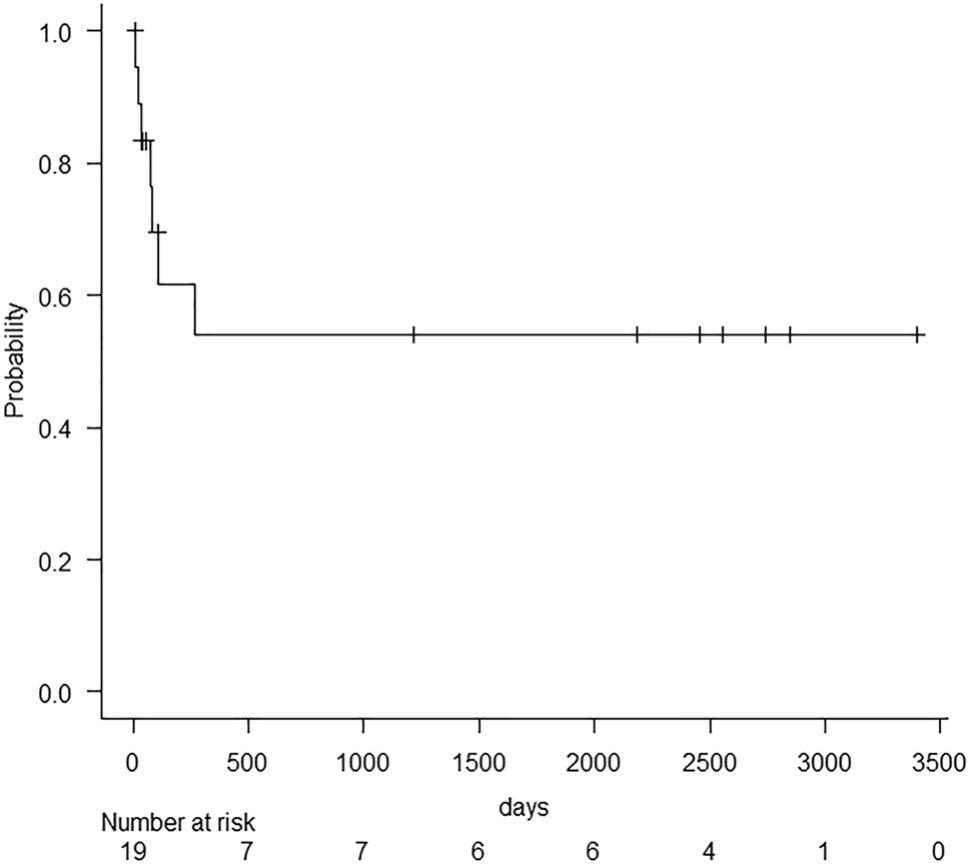
Kaplan–Meier analyses for all-cause mortality

**Fig. 4:**
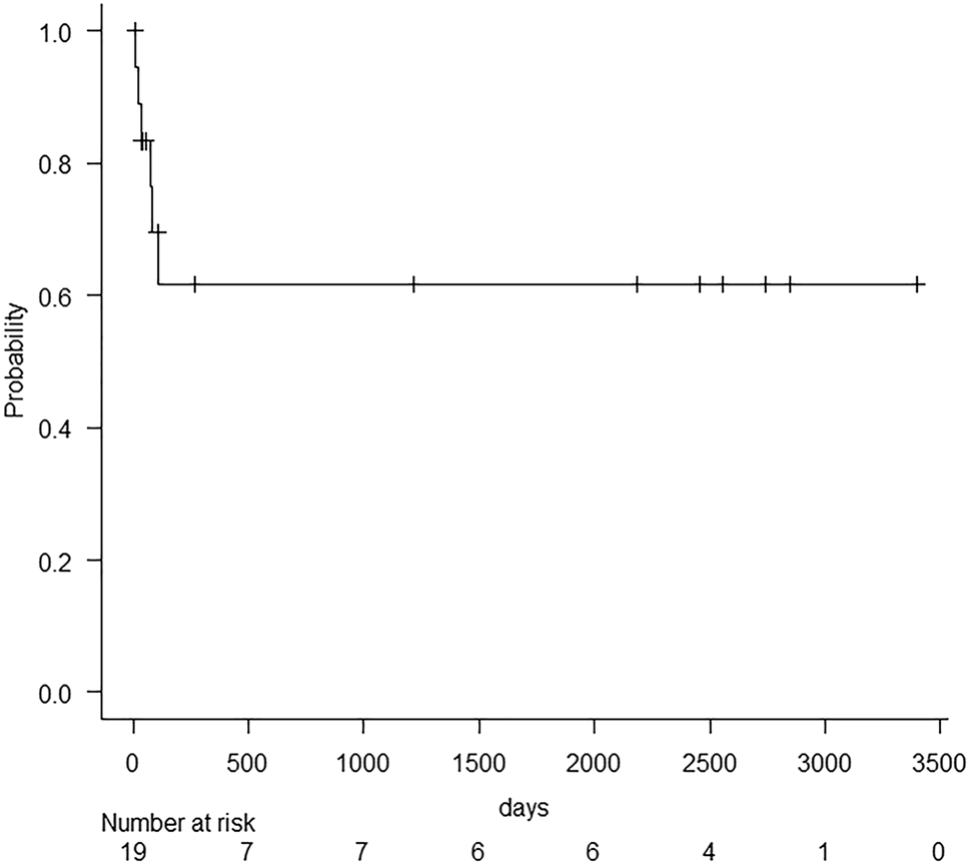
Kaplan–Meier analyses for acute and subacute onset of portal vein and mesenteric vein thrombosis-related mortality

Kaplan–Meier analyses for all-cause mortality and acute–subacute PVMVT-related mortality are depicted in Figs. 3, 4. The average observation period was 960.3 ± 1227.0 (range, 7–3397) days. During this period, seven patients died on days 10, 21, 32, 68, 78, 106, and 267 post-EVTs; death was caused by multiple organ failure in five patients, hemoperitoneum in one patient, and lung carcinoma in one patient. Therefore, the all-cause and acute–subacute PVMVT-related mortality rates were 36.8% (7/19) and 31.6% (6/19), respectively. Seven patients recovered clinically and survived for > 3 years after the intervention. Death due to hemoperitoneum occurred 32 days after EVTs, without thrombolysis and anticoagulation being performed. Hemoperitoneum was caused by disseminated intravascular coagulation related to PVMVT and had no direct relationship with EVTs. The five cases of death due to multiple organ failure were in-hospital deaths, and multiple organ failure was considered related to PVMVT.

### Degree of recanalization

The results of EVT data are summarized in Table 3. Overall, 18 patients (94.7%) achieved complete (*n* = 11, 57.9%) and partial recanalization (*n* = 7, 36.8%). Only one patient with an amoebic liver abscess exhibited no recanalization. Twelve patients were switched to oral anticoagulant therapy after completion of EVTs. Ten patients received warfarin, and two received edoxaban as an oral anticoagulant after EVTs. Six patients with acute–subacute PVMVT-related death could not be switched to oral anticoagulation therapy. One patient refused oral anticoagulation therapy following EVTs.

**Table 3:** Summary of EVT results

Degree of recanalization	
Complete recanalization	11 (57.9%)
Partial recanalization	7 (36.8%)
Unchanged	1 (5.3%)

Complete recanalization: no intravascular material remained, and blood flow was restored

Partial recanalization: the thrombosis was less extensive after intervention than before

Unchanged: no recanalization was observed

*EVT* endovascular treatment

### Complications

Data regarding complications are summarized in Table 4. Seven patients (36.8%) developed complications, with all of them being related to bleeding and requiring various interventions: conservative hemostasis in three patients, transfusion in two patients, transarterial embolization of the hepatic artery in one patient, and transarterial embolization of the lumbar artery in one patient. Therefore, three patients were categorized as having grade I, two as grade II, and two as grade IIIa complications. None of the patients developed complications of grade IIIb or higher, and thus, no patients discontinued EVTs due to bleeding.

**Table 4:** Complications

Clavien–Dindo classification	*N* = 19
I	3 (15.8%)
Subcapsular hematoma	2 (10.5%)
Intrahepatic hematoma	1 (5.3%)
II	2 (10.5%)
Hemoperitoneum	1 (5.3%)
Intrahepatic hematoma	1 (5.3%)
IIIa	2 (10.5%)
Intrahepatic hemorrhage	1 (5.3%)
Lumbar arterial hemorrhage	1 (5.3%)
≥ IIIb	0

### Patency of PV

**Fig. 5:**
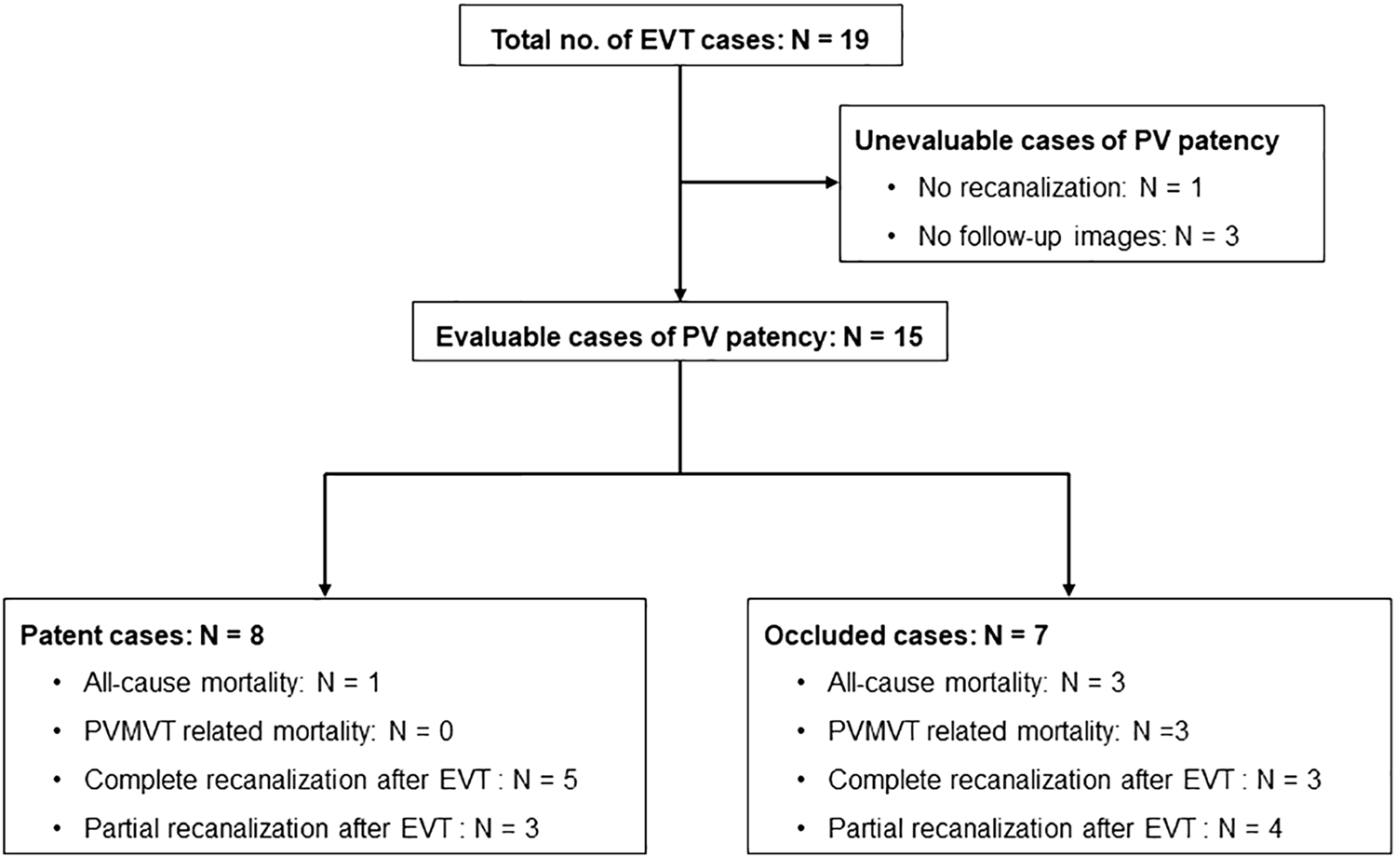
Flowchart of clinical course. *EVT* endovascular treatment, *PV* portal vein, *PVMVT* portal and mesenteric vein thrombosis

**Fig. 6:**
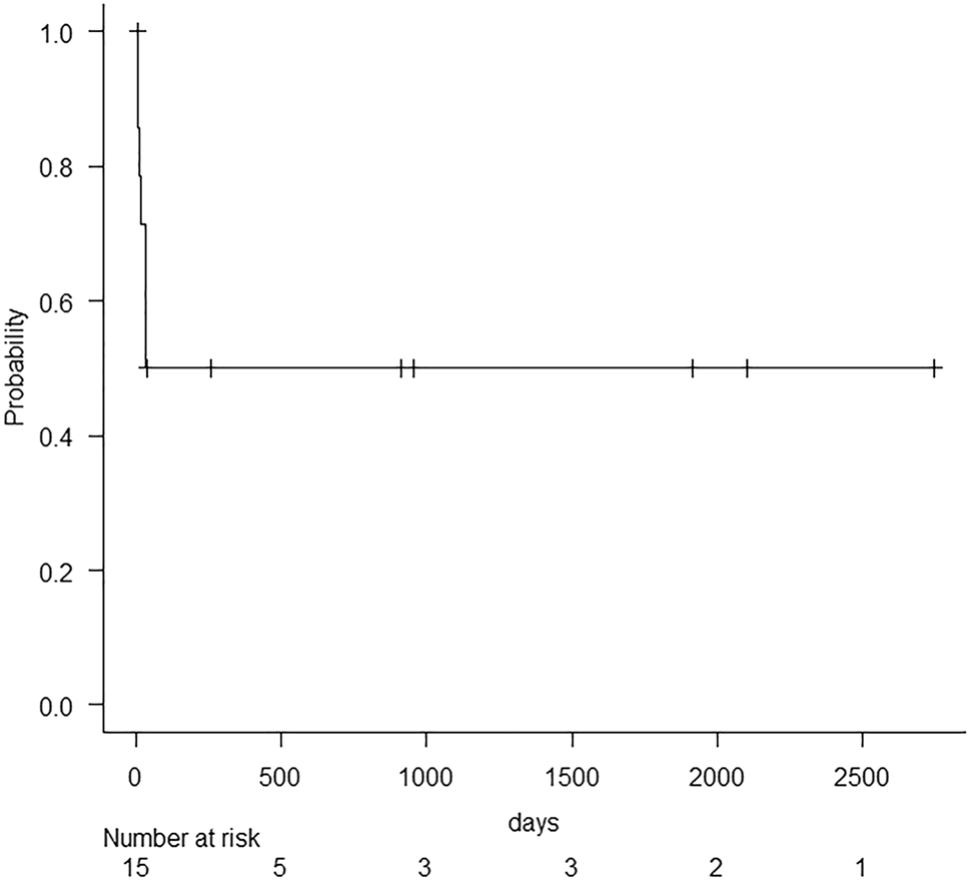
Kaplan–Meier analyses for patency of the portal vein

Figure 5 shows the flowchart of clinical course of patients in this study. In post-EVT follow-ups, CECT or sonography was performed in 15 of the 18 patients who experienced recanalization after EVTs (83.3%), and the mean follow-up period was 608.7 ± 889.5 (range, 2–2743) days. Kaplan–Meier analyses for patency of PV are depicted in Fig. 6. Eight of the 15 patients had complete recanalization, and seven had partial recanalization immediately after EVTs. Eight of the 15 patients (53.3%) achieved patency of PV. Five of these eight patients with complete recanalization and three of the seven patients with partial recanalization achieved patency. The average time to maintain patency after EVTs was 1116.0 ± 965.2 (range, 5–2743) days, and the average time to confirm re-obstruction after EVTs was 20.6 ± 11.2 (range, 6–35) days. However, limited clinical data were available regarding the development of portal hypertension during follow-up.

### Factors related to the PVMVT-related mortality

Table 5 shows factors considered to affect the PVMVT-related mortality. Indication of liver dysfunction was found to be a significant factor related to PVMVT-related mortality (*P* = 0.046). There were no other significant factors related to the PVMVT-related mortality; however, all patients who achieved patency of PV survived during the follow-up period, whereas three of the seven (42.9%) patients whose PVs were occluded died during hospitalization. Moreover, no patients with etiology of PV or SMV stenosis due to surgery died in this study.

**Table 5:** Factors associated with PVMVT-related mortality

Variable	No. of mortality cases	*P* value
Indication		
Bowel ischemia	3 (50.0%)	1
Liver dysfunction	4 (66.7%)	0.046
Expansion of PVMVT	1 (16.7%)	0.18
Etiology		
Biliary and pancreatic fistula	3 (50.0%)	0.26
PV or SMV stenosis by surgery	0 (0.0%)	0.52
Infection	1 (16.7%)	1
Coagulation disorders	2 (33.3%)	1
Idiopathic PVMVT	0 (0.0%)	1
EVT procedure		
Transileocolic approach	1 (16.7%)	1
Stent placement	5 (83.3%)	0.06
Continuous CDT	4 (66.7%)	0.22
Degree of recanalization		
Complete recanalization	4 (66.7%)	1
Partial recanalization	2 (33.3%)	
Unchanged	0 (0.0%)	
Patency of PV		
Patency	0 (0.0%)	0.08
Occlusion	3 (100%)	

*CDT* catheter-directed thrombolysis, *EVT* endovascular treatment, *PV* portal vein, *PVMVT* portal and mesenteric vein thrombosis, *SMV* superior mesenteric vein thrombosis

### Factors related to the patency of PV

Table 6 shows factors considered to affect PV. All patients (100%) who maintained patency of PV were administered post-oral anticoagulant therapy, while two out of seven patients with occlusion (28.6%) were administered post-oral anticoagulant therapy. The percentage of patients with patency who were administered post-oral anticoagulant therapy was significantly higher than that of patients with occlusion (*P* = 0.03). No other factors were significantly related to the patency of PV.

**Table 6:** Factors related to the patency of PV

Variable	No. patent cases	*P* value
Indication		
Bowel ischemia	5 (62.5%)	0.32
Liver dysfunction	1 (12.5%)	0.57
Expansion of PVMVT	3 (37.5%)	0.62
Etiology		
Biliary and pancreatic fistula	1 (12.5%)	1
PV or SMV stenosis by surgery	3 (37.5%)	0.2
Infection	1 (12.5%)	0.28
Coagulation disorders	3 (37.5%)	1
Idiopathic PVMVT	0 (0.0%)	0.47
EVT procedure		
Transileocolic approach	2 (25.0%)	0.47
Stent placement	3 (37.5%)	1
Continuous CDT	7 (87.5%)	1
Degree of recanalization		
Complete recanalization	5 (62.5%)	0.62
Partial recanalization	3 (37.5%)	
Oral anticoagulation	8 (100.0%)	0.03

*CDT* catheter-directed thrombolysis, *EVT* endovascular treatment, *PV* portal vein, *PVMVT* portal and mesenteric vein thrombosis, *SMV* superior mesenteric vein thrombosis

## Discussion

The indications and methods of EVTs have not been established, although the prognosis of severe acute–subacute PVMVT has been reported to be poor even in the recent years [3–8]. Our study showed that the all-cause and acute–subacute PVMVT-related mortality rates were 36.8% (7/19) and 31.6% (6/19), respectively. The mortality rate in our study was high despite the aggressive treatment; however, the treatment outcome was considered acceptable, because the mortality rate of severe acute–subacute PVMVT has been reported to be 50–75% in studies including various degrees of occlusion rates of PV and MV [3–8]. This study showed that deterioration of liver function worsens prognosis. Acute–subacute PVMVT with severe complications, such as bowel ischemia and portal hypertension with variceal hemorrhage, also has a poor prognosis [5–8]; however, there were no other factors that affected the prognosis. Unfortunately, we cannot conclude whether the aggressive treatment employed in our study affected disease progression; however, seven patients recovered clinically and survived for over 3 years after the intervention. Moreover, all patients who achieved patency of PV survived during the follow-up period, whereas 42.9% of patients whose PVs were occluded died during hospitalization. Thus, aggressive intervention might have improved the prognosis.

This study showed that 18 of the 19 patients (94.7%) achieved recanalization, and complications occurred in seven of the 19 patients (36.8%): grade I in three patients, grade II in two patients, and grade IIIa in two patients. Only two patients had complications requiring intervention. A technical success rate of 75–82% has been reported for EVTs [17, 24, 25, 32]; therefore, we achieved a high recanalization rate. In terms of complications, Hollingshead et al. reported that 60% of their patients experienced major complications, and one patient died of gastrointestinal bleeding and sepsis [24]. Alnahhal et al. reported a complication rate of 8.3% [17]. Contrastingly, although the complication rate in our study was high, no procedure-related death occurred, and all complications could be treated with either transfusion or transarterial embolization.

The goal of acute–subacute PVMVT treatment is to reconstruct the blocked veins; however, no reports clearly define the treatment endpoint for PVMVT. The establishment of portal circulation, which refers to hepatoportal blood flow from the PV to the hepatic vein via sinusoids, is important to maintain patency. The establishment of portal circulation in some areas may maintain patency even when only partial recanalization is achieved. EVT procedures, such as AT, POBA, and stent placement, are effective in the removal of thrombi in the main tract of PV and SMV but cannot remove minute peripheral thrombi. Conversely, continuous CDT appears to be effective against minute peripheral thrombi that cannot be removed using EVTs. Therefore, a combination of repetitive thrombectomy and continuous CDT is important for establishing portal circulation. However, sometimes, re-occlusion occurs in the early stage, even if complete radiographic recanalization is achieved. A high rate of re-occlusion has been reported to occur at an early stage [22, 25]. This study showed that only 53.3% of cases achieved patency. This study also showed that anticoagulation therapy after EVTs is important to maintain patency of PV, as previously reported [5, 14]. Conclusive statements regarding any other factors that lead to re-occlusion are not within the scope of this study. However, it has been reported that patients with “idiopathic” mesenteric venous thrombosis (MVT) have the best long-term prognosis, whereas those with an associated inflammatory condition or those whose MVT occurred in the postoperative period have intermediate long-term survival [12].

The percutaneous transhepatic and trans-splenic and transjugular approaches, and transileocolic approaches to PV under laparotomy are known [13, 16, 17, 25]. The percutaneous approach is less invasive than the transileocolic approach [16, 33–35]. The transileocolic approach, which requires a laparotomy and an open abdomen approach with VAC, is limited to cases requiring bowel resection [36]. However, an open abdomen approach with VAC is beneficial for the prevention of abdominal compartment syndrome and observation of further bowel necrosis and hemoperitoneum [28, 37].

Major limitations of this study include the small study sample and the different patient etiologies. It is impossible to determine whether there were patients with symptoms similar to PVMVT who were not referred to our center, and the clinical outcome of the total patients with acute–subacute PVMVT in the surrounding area during the study period is unknown. The devices used were also varied, and multiple operators were involved because of the long study duration, and this lack of uniformity might have affected the outcome.

Our results suggest that the combination of temporary thrombectomy and continuous CDT is considered effective in patients with life-threatening acute–subacute PVMVT. However, further studies must define conditions that improve patient prognosis.
